# Elevation of inositol pyrophosphate IP_7_ in the mammalian spinal cord of amyotrophic lateral sclerosis

**DOI:** 10.3389/fneur.2023.1334004

**Published:** 2024-01-11

**Authors:** Masatoshi Ito, Natsuko Fujii, Saori Kohara, Masayuki Tanaka, Masaki Takao, Ban Mihara, Yuko Saito, Atsushi Mizuma, Taira Nakayama, Shizuka Netsu, Naoto Suzuki, Akiyoshi Kakita, Eiichiro Nagata

**Affiliations:** ^1^Department of Neurology, Tokai University School of Medicine, Isehara, Japan; ^2^Department of Legal Medicine, St. Marianna University School of Medicine, Kawasaki, Japan; ^3^Support Center for Medical Research and Education, Tokai University, Isehara, Japan; ^4^Department of Clinical Laboratory, National Center of Neurology and Psychiatry, National Center Hospital, Tokyo, Japan; ^5^Department of Neurology, Mihara Memorial Hospital, Isesaki, Japan; ^6^Department of Neuropathology, Tokyo Metropolitan Geriatric Hospital and Institute of Gerontology, Tokyo, Japan; ^7^Department of Pathology, Brain Research Institute, Niigata University, Niigata, Japan

**Keywords:** amyotrophic lateral sclerosis, inositol pyrophosphate, diphosphoinositol pentakisphosphate, inositol hexakisphosphate, liquid chromatography-tandem mass spectrometry

## Abstract

**Background:**

Amyotrophic lateral sclerosis (ALS) is a fatal neurodegenerative disorder associated with progressive impairment of spinal motor neurons. Continuous research endeavor is underway to fully understand the molecular mechanisms associating with this disorder. Although several studies have implied the involvement of inositol pyrophosphate IP_7_ in ALS, there is no direct experimental evidence proving this notion. In this study, we analyzed inositol pyrophosphate IP_7_ and its precursor IP_6_ in the mouse and human ALS biological samples to directly assess whether IP_7_ level and/or its metabolism are altered in ALS disease state.

**Methods:**

We used a liquid chromatography-mass spectrometry (LC-MS) protocol originally-designed for mammalian IP_6_ and IP_7_ analysis. We measured the abundance of these molecules in the central nervous system (CNS) of ALS mouse model *SOD1*(G93A) transgenic (TG) mice as well as postmortem spinal cord of ALS patients. Cerebrospinal fluid (CSF) and peripheral blood mononuclear cells (PBMCs) from ALS patients were also analyzed to assess if IP_7_ status in these biofluids is associated with ALS disease state.

**Results:**

*SOD1*(G93A) TG mice showed significant increase of IP_7_ level in the spinal cord compared with control mice at the late stage of disease progression, while its level in cerebrum and cerebellum remains constant. We also observed significantly elevated IP_7_ level and its product-to-precursor ratio (IP_7_/IP_6_) in the postmortem spinal cord of ALS patients, suggesting enhanced enzymatic activity of IP_7_-synthesizing kinases in the human ALS spinal cord. In contrast, human CSF did not contain detectable level of IP_6_ and IP_7_, and neither the IP_7_ level nor the IP_7_/IP_6_ ratio in human PBMCs differentiated ALS patients from age-matched healthy individuals.

**Conclusion:**

By directly analyzing IP_7_ in the CNS of ALS mice and humans, the findings of this study provide direct evidence that IP_7_ level and/or the enzymatic activity of IP_7_-generating kinases IP6Ks are elevated in ALS spinal cord. On the other hand, this study also showed that IP_7_ is not suitable for biofluid-based ALS diagnosis. Further investigation is required to elucidate a role of IP_7_ in ALS pathology and utilize IP_7_ metabolism on the diagnostic application of ALS.

## 1 Introduction

Amyotrophic lateral sclerosis (ALS) is an incurable neurodegenerative disorder categorized to the progressive motor neuron disease, and its incident is most frequent in sexagenarians and septuagenarians (1, 2). An epidemiological study showed that 1.68 per 100,000 person-years suffered from this disease in worldwide with substantial variations in region (3). Sporadic ALS where the ALS onset is independently of hereditary traits accounts for 70–80% of all of the ALS cases, confounding the genetic prediction of future ALS onset (4). Due to the absence of effective biomarkers for ALS, its diagnosis hinges on the indirect approaches by checking clinical symptoms and measuring muscle action potential amplitudes, which results in the delayed therapeutic intervention with limited number of medical options (5). To tackle these issues, a number of ALS-causative proteins including C9orf72, TDP-43 and FUS have been identified and studied for the development of ALS therapeutic agents selectively targeting these molecules (6, 7). In addition, the discovery of potentially diagnostic biomarkers for ALS such as Neurofilament L (NfL) facilitates the biofluid-based noninvasive approaches for ALS diagnosis (8–10). Yet, there is still a lack of molecular information with regard to the ALS pathology, and thus its features in molecular machinery should be elucidated more clearly for liberating humanity from the agony of this disease.

Inositol pyrophosphate exists in a wide variety of organisms from slime molds and fungi to mammals and is involved in numerous cellular processes including intracellular signaling (11–13). Diphosphoinositol pentakisphosphate (a.k.a. IP_7_), a representative inositol pyrophosphate in mammals, is synthesized from the precursor molecule inositol hexakisphosphate (a.k.a. IP_6_) by inositol hexakisphosphate kinases (IP6Ks). So far, several lines of evidence suggested pathological roles of IP6Ks and IP_7_ in stress response and neurodegeneration. IP6K2, one of the major IP_7_-synthesizing kinase in mammals, was characterized as a cell death mediator (14) and IP_7_ facilitates cellular apoptosis by reactive oxygen treatment (15). Considering our previous observations of IP6K2 mRNA induction during presymptomatic disease state of ALS (16) and IP6K2 modulatory role in TDP-43-mediated cellular apoptosis (17), these facts collectively imply the notion that IP_7_ would be induced in aberrant motor neuron of ALS disease state. However, none of the direct evidences has not been obtained due to lack of technologies directly detecting and quantifying IP_7_. Recently, we developed an analytical protocol directly and selectively detecting IP_7_ in mammalian tissues (18, 19), unlocking the direct evaluation of this molecule in various clinical biopsies.

In this study, we analyzed endogenous IP_7_ and its precursor IP_6_ in ALS model mice as well as human ALS patients by an originally-designed liquid chromatography mass spectrometry (LC-MS) protocol and assessed if ALS disease state would be accompanied by altered IP_7_ level.

## 2 Materials and methods

### 2.1 Human samples and peripheral blood cell fractionation

Frozen postmortem spinal cords derived from 9 ALS patients and five neurologically normal patients were obtained from Japan Brain Bank Net (JBBN) and Brain Research Institute of Niigata University. Each 3 mL of cerebrospinal fluid (CSF) was collected from 3 ALS patients by lumber puncture. Peripheral blood samples (20 mL) were collected from 25 ALS patients after the definitive diagnosis by the El Escorial diagnostic criteria (20) and 22 age-matched healthy controls in our hospital (Supplementary Tables 1, 2). Three out of 25 ALS patients were excluded as statistical outliers, and therefore 22 ALS patients were used for the subsequent analysis. After the isolation using a Lymphocyte Separation Solution (Nacalai Tesque, Japan), peripheral blood mononuclear cells (PBMCs) were lysed by Lysis buffer (0.01% Triton X-100, 1 mM EDTA, 20 mM Tris-HCl). After centrifugation, the supernatant was further processed to isolate IP_6_ and IP_7_ for the subsequent LC-MS analysis. All participants before passing away or their families provided written informed consent. Experiments using human samples were performed with institutional approval and guidelines from the Clinical Investigation Committee at Tokai University School of Medicine (institutional review board No. 10R-010).

### 2.2 Mouse samples

All experiments involving animals were performed in accordance with protocols approved by institutional animal care guidelines (Tokai University School of Medicine). *SOD1*(G93A) transgenic (TG) mice and littermate wild-type (WT) mice were obtained from Clea Japan (Tokyo, Japan) and maintained at an ambient temperature of 23 ± 2°C and humidity of 55 ± 15% with a 12 h light-dark cycle. Food (CE-2; Clea Japan) and water were fed *ad libitum*. The behavioral performance of the TG mice was regularly monitored by rotarod test. These mice were anesthetized using isoflurane and then sacrificed to collect the central nervous system (CNS; cerebrum, cerebellum, spinal cord). The harvested organs were frozen until further use.

### 2.3 Extraction of IP_6_ and IP_7_ from human and mouse samples

Human and mouse frozen tissues were homogenized using a Shake Master Neo (Bio Medical Science). The crude lysates were centrifuged to collect the supernatants. The supernatants from tissues and cells were mixed with an equal volume of 2 M perchloric acid and further centrifuged to remove insoluble protein fraction. After adding 3 nmol of hexadeutero-myo-inositol trispyrophosphate (ITPP-d_6_, Toronto Research Chemicals) as a surrogate internal standard, IP_6_ and IP_7_ were purified using titanium dioxide beads (GL Sciences) as described previously (21).

### 2.4 Measurements of IP_6_ and IP_7_ by liquid chromatography–tandem mass spectrometry (LC-MS)

Quantitative measurement of IP_6_ and IP_7_ in human and mouse samples were performed using an originally-designed LC-MS protocol (18, 19). Briefly, chromatographic separation of IP_6_, IP_7_, and internal standard ITPP-d_6_ is achieved by hydrophilic interaction liquid chromatography (HILIC) mode with a polymer-based bioinert column (HILICpak VG-50 2D; Shodex, Tokyo, Japan). The aqueous mobile phase was 300 mM ammonium bicarbonate buffer (pH 10.5) containing 0.1% InfinityLab deactivator additive (Agilent Technologies) and the organic mobile phase was 90% acetonitrile containing 10 mM ammonium bicarbonate buffer (pH 10.5) and 0.1% InfinityLab deactivator additive. The total flow rate of the mobile phase was 0.4 ml/min. Linear gradient separation was achieved as follows: 0–2 min, 75% B; 2–12 min, 75%−2% B; 12–15 min, 2% B. Mass spectrometric detection of these molecules was performed by selected reaction monitoring (SRM) using a LCMS-8050 triple quadrupole mass analyzer (Shimadzu corporation, Kyoto, Japan).

### 2.5 ALSFRS-R scoring

The ALSFRS-R scores of 25 ALS patients whom the peripheral blood was collected from were assessed by two neurologists. ALSFRS-R consists of 12 categories including speaking, eating, and respiratory ability, each of which is scored between 0 and 4 points. Scores decrease along with increasing functional exacerbation, and thus the total ALSFRS-R scores of ALS patients with normal and the worst functional status sum up as 48 (maximum) and 0 (minimum) points, respectively.

### 2.6 Statistical analysis

Statistical analysis was performed by SPSS software ver.26. Data are expressed as the mean ± standard deviation. Differences between two or more groups were analyzed using two-tailed Student's *t*-test. Statistical significance was set at *p* < 0.05. Variable PBMC data of ALS patients were processed by SIMCA-P software (Umetrics, Umeå, Sweden) for the evaluation of statistical outliers.

## 3 Results

### 3.1 Elevated IP_7_ level in the spinal cord of ALS model mice

**Figure 1:**
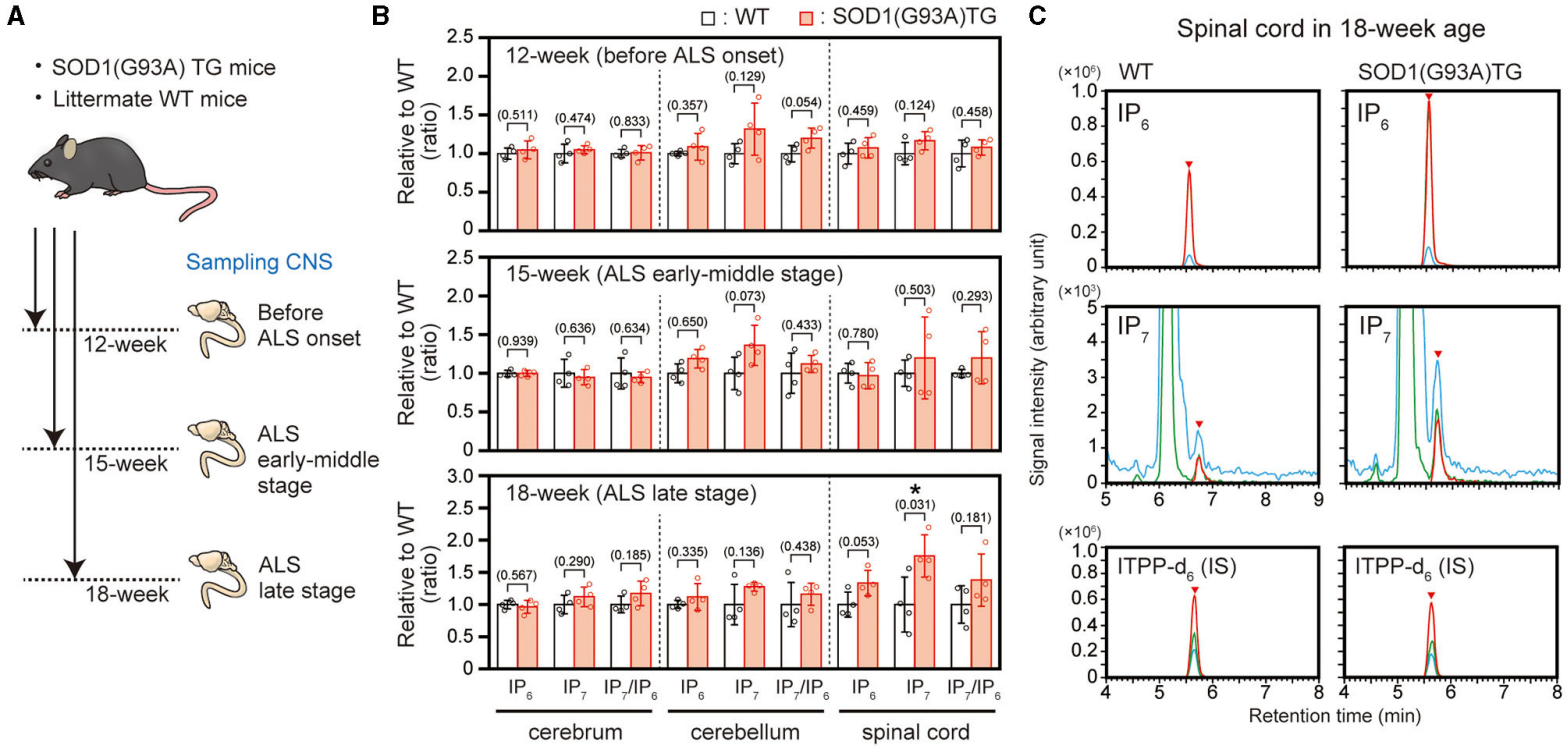
Elevated IP_7_ level in the spinal cord of SOD1(G93A) TG mice in the ALS late stage. **(A)** Schematic illustration of the experimental workflow. SOD1(G93A) TG and their littermate WT mice at 12-week (before ALS onset), 15-week (ALS early-middle stage) and 18-week (ALS late stage) were sacrificed to harvest central nervous system (CNS) for LC-MS analysis. **(B)** The concentrations of IP_6_, IP_7_, and IP_7_/IP_6_ (product-to-precursor) ratio in the cerebrum, cerebellum and spinal cord of SOD1(G93A) TG and their littermate WT mice. The values shown represent the mean ± SD of four independent experiments and are expressed relative to the WT mice. *P*-values calculated by Student's *t*-test are given in parenthesis. Asterisks indicate statistical significance (*p* < 0.05) compared with WT mice. **(C)** Representative SRM chromatograms of IP_6_, IP_7_, and internal standard ITPP-d_6_ in the spinal cord of SOD1(G93A) TG and their littermate WT mice. The arrowheads indicate the SRM peaks of the corresponding analytes. IS, internal standard.

We previously showed that IP6K2 mRNA level was increased in the spinal cord of ALS patients, implying elevated production of IP_7_ in the ALS spinal cord (16, 17). To verify this hypothesis, we analyzed IP_7_ and its precursor IP_6_ in the CNS of ALS mouse model *SOD1*(G93A) transgenic (TG) mice at the three different breeding points, namely 12-week age (before ALS onset), 15-week age (early-middle stage of ALS), and 18-week age (late stage of ALS; Figure 1A). These TG mice become impairing their motor activity around 16-week age and almost completely lose lower limb mobility with the moribund state around 18-week age, which is in accordance with a previous report (22). LC-MS analysis showed that IP_7_ level was significantly increased in the spinal cord of the TG mice compared with that of littermate control mice at 18-week age (late stage of ALS), while its level did not significantly change in cerebrum and cerebellum of the same mice (Figures 1B, C). In the spinal cord of TG mice at 18-week age, IP_6_ level was also increased slightly but not significantly. Thus, we observed elevated IP_7_ level in the spinal cord of a rodent ALS model at ALS progressive state.

### 3.2 Elevated IP_7_ level in the postmortem spinal cord of ALS patients

**Figure 2:**
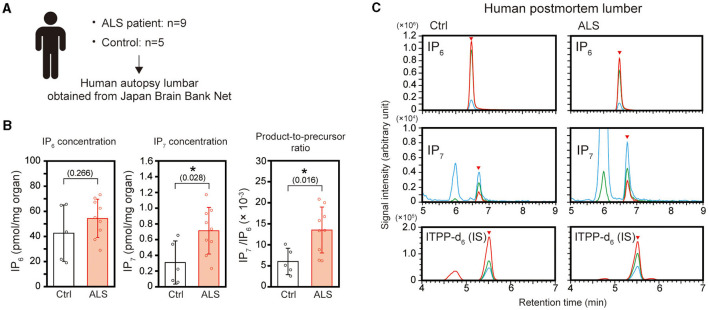
Elevation of IP_7_ level and its production rate in the postmortem lumber cord of ALS patients. **(A)** Schematic depiction of the experimental workflow. Human postmortem lumber cords of ALS patients (*n* = 9) and neurologically normal patients (control; *n* = 5) obtained from Japan Brain Bank Net were subjected to LC-MS analysis. **(B)** The concentration of IP_6_ (left panel), IP_7_ (middle panel), and IP_7_/IP_6_ (product-to-precursor) ratio (right panel) in the postmortem lumber cords of ALS patients and neurologically normal patients (controls). The values shown represent the mean ± SD of nine (ALS) and five (control) independent experiments. *P*-values calculated by Student's *t*-test are given in parenthesis. Asterisks indicate statistical significance (*p* < 0.05) compared with the controls. **(C)** Representative SRM chromatograms of IP_6_, IP_7_, and internal standard ITPP-d_6_ in the postmortem lumber of ALS patients and controls. The arrowheads indicate the SRM peaks of the corresponding analytes. Ctrl, control; IS, internal standard.

To further confirm the elevated IP_7_ induction in the ALS spinal cord, we prepared 9 and 5 biopsies of human postmortem lumber cord from ALS patients and neurologically normal patients (control), respectively (Figure 2A and Table 1). The average ages of these two groups were 66.0 ± 9.96 for ALS patients and 70.8 ± 1.79 for control patients. All ALS patients examined were at the late stage of the disease and died by respiratory failure manifested as ALS-related dysfunction. IP_7_ level and the product-to-precursor (IP_7_/IP_6_) ratio were significantly increased in the postmortem lumber cord of the ALS patients compared with that of controls, while IP_6_ level was comparable between these two groups (Figures 2B, C). Thus, we demonstrated that IP_7_ level and its production rate are significantly elevated in the human spinal cord during ALS disease state.

**Table 1:** Details of deceased patients donated their spinal cord in this study.

	**Individual no**.	**Sex**	**Age (year)**	**Duration of illness (year)**	**Predominant clinical feature of ALS**	**Cause of death**	**Respirator**
ALS	#1	M	53	5	UL	RF	–
	#2	M	59	4	LL	RF	+
	#3	M	58	1	LL	RF	–
	#4	F	80	2	LL	RF	+
	#5	M	67	10	UL, LL	RF	+
	#6	F	57	14	UL	RF	+
	#7	M	74	2	UL	RF	–
	#8	M	67	2	B	RF	–
	#9	F	79	1	B	RF	–
Control	#1	M	71	–	–	Pancreatic cancer	–
	#2	F	71	–	–	Sepsis	+
	#3	F	73	–	–	Pneumonia	–
	#4	M	71	–	–	Pneumonia	–
	#5	M	68	–	–	Pneumonia	–

M, male; F, female; UL, upper limb; LL, lower limb; B, bulbar; RF, respiratory failure.

### 3.3 IP_7_ is not suitable for usage as biofluid-based biomarker for ALS diagnosis

**Figure 3:**
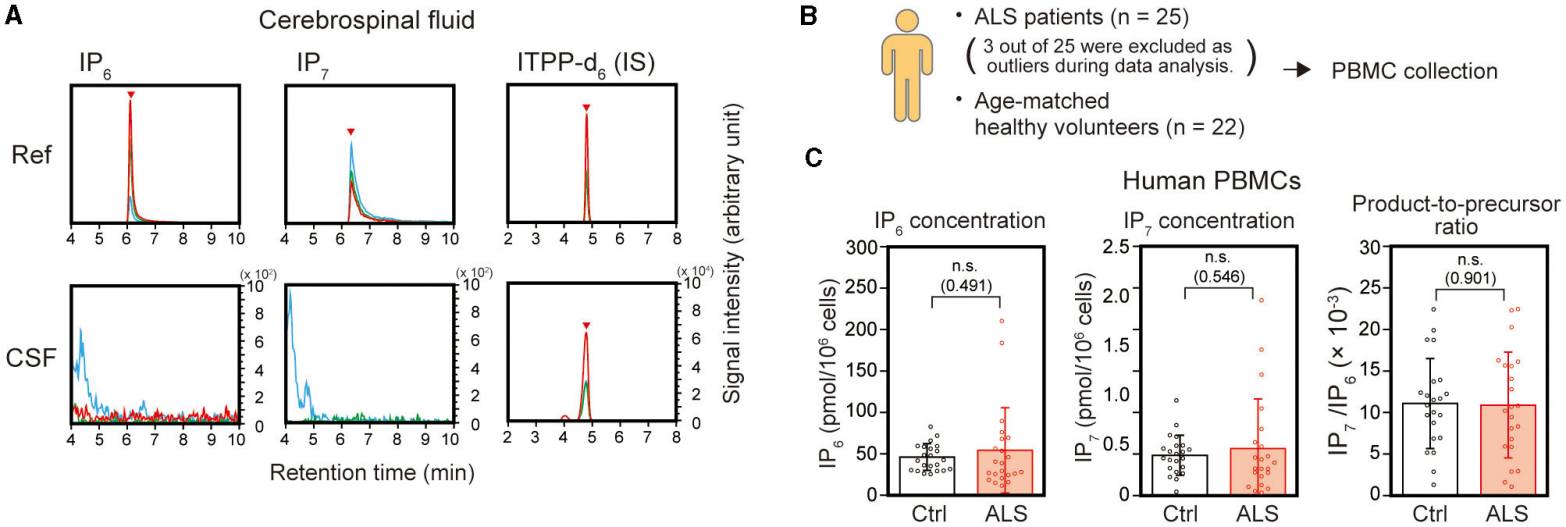
IP_7_ level in human CSF and PBMCs do not differentiate ALS patients from healthy controls. **(A)** Representative SRM chromatogram of IP_6_ (left panels), IP_7_ (middle panels), and internal standard (IS) ITPP-d_6_ (right panels) in the CSF of ALS patients. Three mL of CSF was used for this analysis. SRM peaks of these molecules obtained by analyzing these standards were shown as reference. The arrowheads indicate the SRM peaks of the corresponding analytes. Neither IP_6_ nor IP_7_ were detected in any of the human CSF collected in this study. **(B)** Schematic illustration of the experimental workflow. Twenty milliliters of peripheral blood was collected from ALS patients (*n* = 25) and age-matched volunteers without any neurological disorder (control; *n* = 22). Harvested PBMCs were processed to isolate IP_6_ and IP_7_, which were analyzed by LC-MS. **(C)** The concentration of IP_6_ (left panel), IP_7_ (middle panel), and IP_7_/IP_6_ (product-to-precursor) ratio (right panel) in the PBMCs of ALS patients and controls. The values shown represent the mean ± SD of each 22 independent experiments. *P*-values calculated by Student's *t*-test are given in parenthesis. Ref, reference; Ctrl, control; IS, internal standard; n.s., not significant.

Certain ALS-associated proteins such as NfL and TDP-43 exist in biofluids and are considered as promising biofluid-based biomarkers for ALS diagnosis (23). To assess the availability of IP_7_ as a biofluid-based diagnostic marker, we first analyzed CSF of 3 ALS patients. However, neither IP_7_ nor IP_6_ were detected in CSF samples (Figure 3A). We next attempted to analyze peripheral blood because our and other groups have shown that certain amount of IP_7_ is present in the human peripheral blood (18, 24, 25). By fractionation of human peripheral blood into cell subsets and plasma, we found that peripheral blood mononuclear cells (PBMCs) predominantly possess both IP_6_ and IP_7_ among major fractions of peripheral blood (Supplementary Figure 1). We analyzed the PBMCs of 25 ALS patients and excluded three of them as statistical outliers based on IP_6_ and IP_7_ levels, IP_7_/IP_6_ ratio, and ALSFRS-R values (Supplementary Figure 2). We next compared the levels of IP_6_, IP_7_, and IP_7_/IP_6_ ratio in the PBMCs of ALS patients (*n* = 22) with those in the age-matched healthy counterparts (*n* = 22; Figure 3B). The level of IP_6_, IP_7_, and IP_7_/IP_6_ ratio in the PBMCs were comparable between ALS patients and age-matched healthy counterparts (Figure 3C). Also, these values did not show significant correlations with ALSFRS-R (Supplementary Figure 3). Thus, we failed to suggest that IP_7_ can be used as an ALS biomarker using biofluid such as CSF and peripheral blood.

## 4 Discussion

While a number of studies have identified causative proteins and potential biomarkers for ALS so far (6), such exploring efforts still continue to understand the molecular machinery of this disease more precisely. Several studies reported the implicit findings that inositol pyrophosphate IP_7_ might be associated with ALS pathogenesis (14–17), but there has been no direct evidence proving this notion. In this study, we analyzed IP_7_ and its precursor IP_6_ in ALS model mice and ALS patients by an originally-designed LC-MS protocol (18, 19) to directly examine the relationship between IP_7_ and ALS.

We used a canonical ALS mouse model *SOD1*(G93A) TG mice to analyze IP_7_ level and metabolism before and after ALS onset and found that IP_7_ level significantly increased in the spinal cord of the TG mice at the late stage of the disease (Figures 1B, C). The spinal cord of the TG mice showed slightly but not significantly elevated IP_7_ metabolism (IP_7_/IP_6_ ratio) due to the up-regulation of IP_6_ level concomitantly with IP_7_ elevation, implying dysregulation of the metabolic pathway in lower inositol phosphates (IPs). A recent transcriptome analysis using the TG mice exhibited that mRNA levels of certain genes involved in phosphatidyl inositol metabolic process such as INPP5D (inositol polyphosphate-5-phosphatase D) and INPPL1 (inositol polyphosphate phosphatase like 1) was changed in the spinal cord at ALS disease state (26). Such alteration in inositol phospholipid pathway might lead to the dysregulated metabolism of IP_7_ and other lower inositol phosphates. Similar with the results of this rodent ALS model, significant elevation of IP_7_ level and metabolism was observed in the postmortem lumber of human ALS patients (Figures 2B, C). Considering our previous data that the transcript level of IP_7_-synthesizing enzyme IP6K2 increased in the spinal cord of ALS disease state (16), transcriptional activation of this enzyme is likely to contributes to IP_7_ elevation in the spinal cord of ALS.

The molecular mechanism underlying IP_7_ induction in the ALS condition is still elusive, but IP_7_ and its synthesizing kinase IP6Ks has been shown to associate with several neurodegeneration-related proteins. In our previous report, IP6K2 interacts with TDP-43 and promotes TDP-43-inducing cell death (17). A recent study identified IP_7_ kinase PPIP5K as an α-synuclein neurotoxicity modulator by functional RNAi screening using nematode Perkinson's disease model (27). Moreover, IP6K and IP_7_ facilitate the formation of aberrant protein-RNA aggregates inducing neurotoxicity in various neurodegenerative disorders (28) at least via promoting the interaction of RNA-binding proteins (for IP6K) and inhibiting the 5′-decapping reaction of non-translated mRNAs (for IP_7_) (29, 30). In addition, IP_7_ competitively binds to AKT and inhibits its downstream signaling including mTOR, a key regulator of cell survival (31, 32) (Information of proteins associating with IP_6_ was summarized in Supplementary Figure 4). These pieces of knowledge suggest that IP_7_ could regulate various neurodegenerative-related proteins in a multifaceted manner. However, we did not investigate pathobiological role of IP_7_ in the CNS of ALS model mice because the major barrier for studying IP_7_ functions in mouse models is the difficulty to efficiently inhibit IP_7_ production *in vivo*. Two genes IP6K1 and IP6K2 are responsible for IP_7_ production, but the deletion of both genes results in embryonic death and the deletion of single gene partially inhibits its production in the CNS as shown in our recent report (19). Recent report showed that a novel IP6K inhibitor efficiently blocks IP_7_ production *in vivo* (24), which will enable to investigate the role of IP_7_ in ALS using disease model mice. Further investigation will be warranted to prove the notion that elevated IP_7_ is associated with progressive degeneration of spinal motor neurons during ALS.

Since several ALS-associated molecules such as NfL and TDP-43 are promising for applying biofluid-based ALS diagnostic markers (23), we examined the applicability of IP_7_ in such diagnostic approach using peripheral blood and CSF. Among human peripheral blood fractions, PBMCs possessed most abundant IP_6_ and IP_7_ (Supplementary Figure 1), but the levels of these molecules in PBMCs could not differentiate ALS patients from age-matched healthy individuals (Figure 3C), suggesting the unfeasibility of peripheral blood IP_7_ for usage as an ALS diagnostic marker. In addition, our LC-MS analysis could not detect IP_7_ and IP_6_ in human CSF, suggesting that the abundances of these molecules in CSF are less than 0.3 μM considering the lower limit of detection in our LC-MS protocol (18, 19). It would be necessary to evaluate CSF IP_7_ level by more sensitive protocol such as capillary electrophoresis-mass spectrometry (CE-MS) (33) or develop a technology whereby spinal cord IP_7_ is measured non-invasively for considering the application of IP_7_ for ALS diagnosis.

Although this study focused at the relationship between IP_7_ and ALS in this study, it is meaningful to elucidate if IP_7_ level and its metabolism would be altered in other neurodegenerative disorders. So far, IP_7_ has been implied to be associated with certain neurodegenerative diseases such as Huntington's disease (34) and Alzheimer's disease (35). Sensitive mass spectrometric analysis of IP_7_ using clinical biopsies as well as mouse disease models would unveil a pathobiological role of IP_7_ in such diseases in future.

Recently, CE-MS analysis clarified that several IP_7_ isotypes such as 4/6-IP_7_ and 1/3-IP_7_ constitute mammalian IP_7_ in addition to 5-IP_7_, an already-known isotype of mammalian IP_7_ (25). Due to the technical limitation of LC-MS, we could not determine if the IP_7_ status would be affected during ALS at the level of isotype. Further investigation is required which isotypes of IP_7_ is elevated in the spinal cord of ALS disease state.

We demonstrate for the first time that IP_7_ level and/or its metabolism (IP_7_/IP_6_ ratio) were significantly elevated in mouse and/or human ALS spinal cord compared with neurologically normal counterparts in a direct way. On the other hand, this study showed several limitations. First, the sample number of postmortem ALS spinal cord isn't necessarily sufficient for the statistical analysis. Secondly, we failed to demonstrate IP_7_ level and its metabolism as biofluid-based diagnostic parameters for ALS. Lastly, we could not clarify the molecular basis and pathophysiological significance of IP_7_ elevation during ALS disease state. We believe that further investigation of IP_7_ function in ALS pathology will lead to the development of novel diagnostic and therapeutic approach for this incurable disease.

## Data availability statement

The original contributions presented in the study are included in the article/Supplementary material, further inquiries can be directed to the corresponding author/s.

## Ethics statement

The studies involving humans were approved by the Clinical Investigation Committee at Tokai University School of Medicine. The studies were conducted in accordance with the local legislation and institutional requirements. The participants provided their written informed consent to participate in this study. The animal study was approved by the Institutional Animal Care and Use Committee at Tokai University. The study was conducted in accordance with the local legislation and institutional requirements. Written informed consent was obtained from the individual(s) for the publication of any potentially identifiable images or data included in this article.

## Author contributions

MI: Formal analysis, Investigation, Methodology, Visualization, Writing—original draft, Writing—review & editing. NF: Investigation, Writing—review & editing. SK: Investigation, Writing—review & editing. MTan: Formal analysis, Writing—review & editing. MTak: Funding acquisition, Resources, Writing—review & editing. BM: Resources, Writing—review & editing. YS: Funding acquisition, Resources, Writing—review & editing. AM: Writing—review & editing. TN: Writing—review & editing. SN: Writing—review & editing. NS: Writing—review & editing. AK: Funding acquisition, Resources, Writing—review & editing. EN: Conceptualization, Funding acquisition, Project administration, Supervision, Writing—review & editing.
