# Supplementary material for: Elevation of inositol pyrophosphate IP7 in the mammalian spinal cord of amyotrophic lateral sclerosis
Source: Front Neurol. 2024 Jan 11;14:1334004. doi: 10.3389/fneur.2023.1334004 (PMC10808411; doi:10.3389/fneur.2023.1334004)
Supplement: Supplementary file 1 [file Data_Sheet_1.pdf]

Elevation of inositol pyrophosphate IP_7 in the mammalian spinal cord of amyotrophic lateral sclerosis

Masatoshi Ito[1,2,*], Natsuko Fujii[1], Saori Kohara[1], Masayuki Tanaka[3], Masaki Takao[4,5], Ban Mihara[5], Yuko Saito[6], Atsushi Mizuma[1], Taira Nakayama[1], Shizuka Netsu[1], Naoto Suzuki[1], Akiyoshi Kakita[7], Eiichiro Nagata[1,*]

[1]Department of Neurology, Tokai University School of Medicine, Isehara, Japan
[2]Department of Legal Medicine, St. Marianna University School of Medicine, Kawasaki, Japan.
[3]Support Center for Medical Research and Education, Tokai University, Isehara, Japan
[4]Department of Clinical Laboratory, National Center of Neurology and Psychiatry, National Center Hospital, Tokyo, Japan.
[5]Department of Neurology, Mihara Memorial Hospital, Isesaki, Japan
[6]Department of Neuropathology, Tokyo Metropolitan Geriatric Hospital and Institute of Gerontology, Tokyo, Japan
[7]Department of Pathology, Brain Research Institute, Niigata University, Niigata, Japan

*To whom correspondence should be addressed:
Eiichiro Nagata: Department of Neurology, Tokai University School of Medicine, 143 Shimo-Kasuya, Isehara, Kanagawa 259-1193, Japan; enagata@is.icc.u-tokai.ac.jp; Tel: +81-463-93-1121 (ext. 2245); Fax: +81-463-94-8764

Masatoshi Ito: Department of Legal Medicine, St. Marianna University School of Medicine, 2-16-1 Sugao, Miyamae Ward, Kawasaki, Kanagawa 216-8511, Japan; masatoshi.ito@marianna-u.ac.jp; Tel: +81-44-977-8111 (ext. 3556); Fax: +81-44-977-3902

Key words: amyotrophic lateral sclerosis, inositol pyrophosphate, diphosphoinositol pentakisphosphate, inositol hexakisphosphate, liquid chromatography-tandem mass spectrometry

A IP_6 and IP_7 distribution in human peripheral blood

Blood fraction	IP_6 (%)	IP_7 (%)
mononuclear cell	65.0%	99.6%
erythrocyte	34.3%	0%
platelet	0.7%	0.4%
plasma	0%	0%

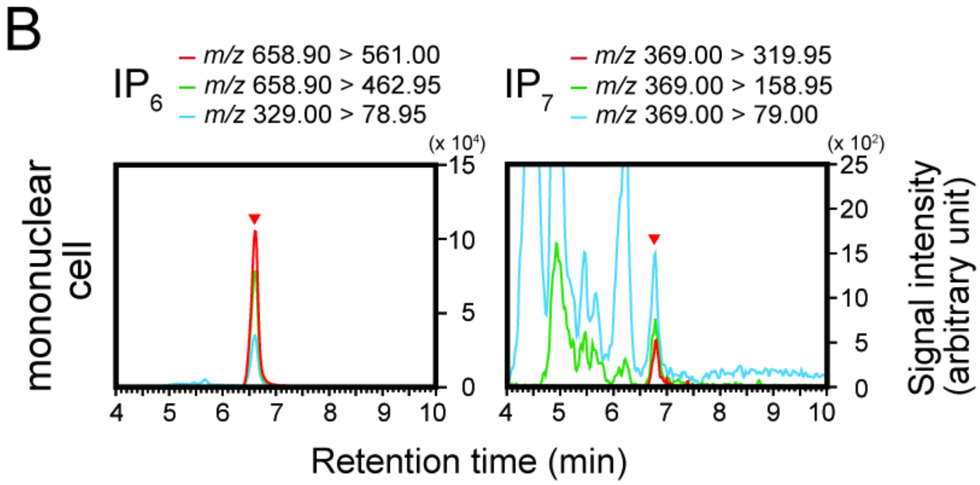

Supplementary Figure 1. IP_6 and IP_7 predominantly exist in the mononuclear cells among human peripheral blood fractions

(A) Quantitative distribution of IP_6 and IP_7 among human peripheral blood fractions. Each value indicates percent distribution of IP_6 and IP_7 levels in the corresponding fractions contained in 1 mL human peripheral blood.

(B) Representative SRM chromatograms of IP_6 (left) and IP_7 (right) in human PBMCs. The arrowheads indicate the SRM peaks of the corresponding analytes.

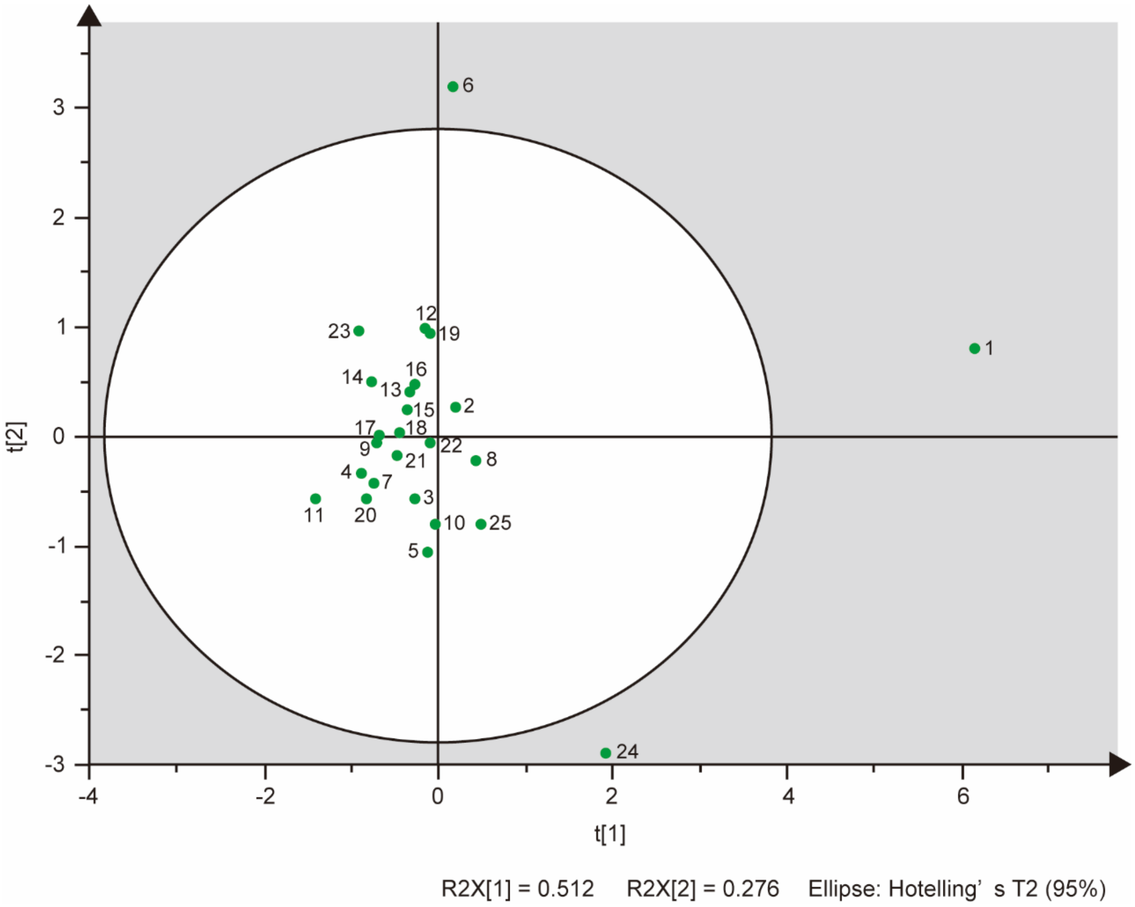

Supplementary Figure 2. Multivariate analysis for the levels of IP_6 and IP_7 levels, IP_7/IP_6 ratios and ALSFRS-R values in the PBMCs of ALS patients

The PCA plot representing t[1] vs. t[2] was depicted. Open circle indicates the boundary of 95% confidence interval. Data of 3 ALS patients (No. 1, 6 and 24) were located outside of the acceptance area and thereby regarded as outliers.

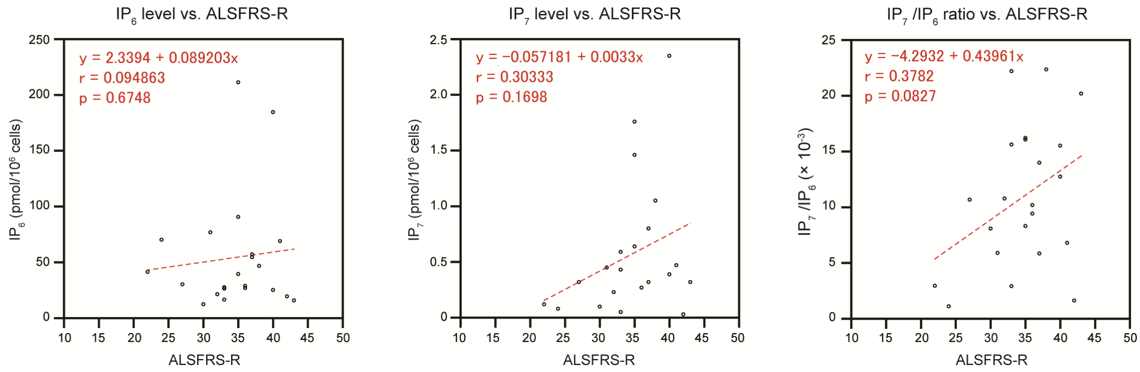

Supplementary Figure 3. IP_6 level, IP_7 level and IP_7/IP_6 ratio in the PBMCs of ALS patients did not correlate with their ALSFRS-R values

Scatter plots of ALSFRS-R and IP_6 level (left panel), IP_7 level (middle panel), and IP_7/IP_6 ratio (right panel) are shown. The dashed lines represent linear regression line. p values and the Pearson's correlation coefficients (r) are indicated. Any regression lines did not show statistical significance ($p < 0.05$).

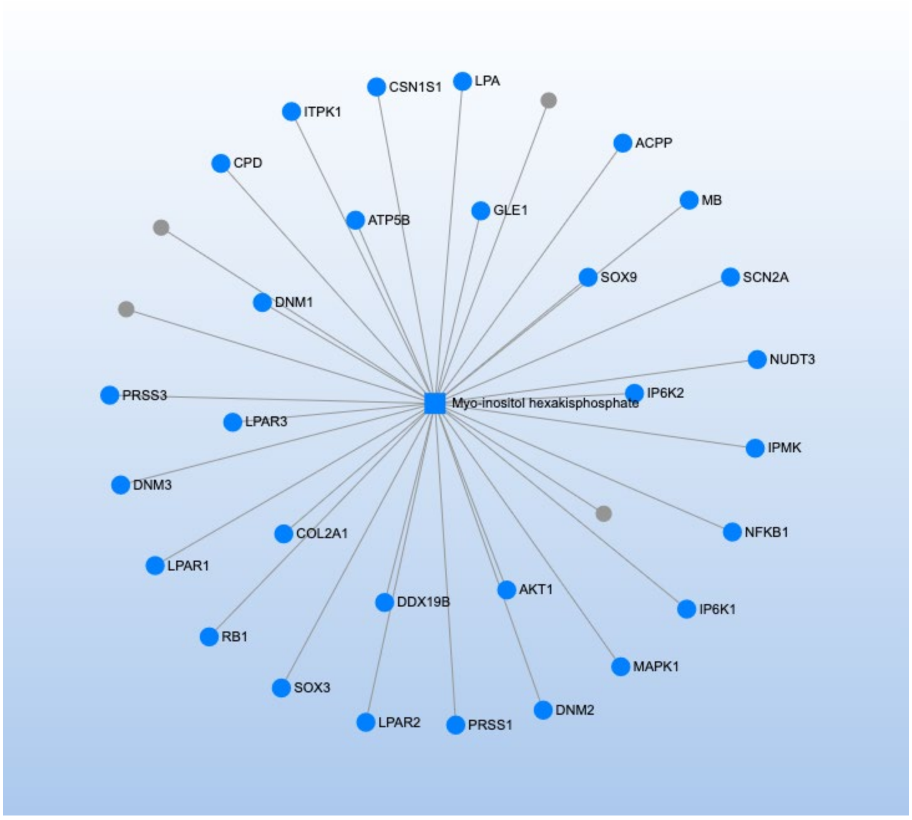

Supplementary Figure 4. Molecular interactions between IP_6 and proteins

IP_6 (center) and its interacting proteins (around the circle) were shown, which is created by MetaboAnalyst 5.0 (https://www.metaboanalyst.ca/). Some of these proteins such as IP6K1, IP6K2, NUDT3 and AKT1 are also known to associate with IP_7.

Supplementary Table 1. Details of ALS patients for peripheral blood analysis in this study

Individual No.	Sex	Age (year)	Duration of illness (year)	Predominant clinical feature of ALS	Number of PBMCs (× 10^6 cells/mL blood)	IP_6 level (pmol/10^6 cells)	IP_7 level (pmol/10^6 cells)	IP_7/IP_6 ratio (× 10^{-3})	ALSFRS-R	Remark
#1	M	71	10	UL	1.50	269.8	8.68	32.16	0	Outlier
#2	F	87	1	B	1.20	90.6	1.46	16.07	35	
#3	F	54	1	LL	1.16	76.9	0.45	5.91	31	
#4	M	67	2	UL	2.31	69.0	0.47	6.80	41	
#5	F	79	1	UL, B	1.39	70.2	0.08	1.12	24	
#6	M	76	1	B	1.77	21.6	1.05	48.33	42	Outlier
#7	F	73	1	UL	1.65	54.6	0.32	5.86	37	
#8	F	64	1	UL	2.06	184.5	2.35	12.75	40	
#9	M	81	2	B	1.92	28.8	0.27	9.43	36	
#10	F	67	0.9	B	1.98	41.3	0.12	2.95	22	
#11	M	51	1	UL, LL	1.46	19.4	0.03	1.64	42	
#12	M	58	1	LL, B	2.83	46.8	1.05	22.37	38	
#13	M	71	1.5	B	2.57	27.7	0.43	15.62	33	
#14	M	73	1	B	2.18	25.2	0.39	15.53	40	
#15	F	77	2	LL	1.83	57.1	0.80	14.00	37	
#16	F	69	0.5	B	1.97	39.4	0.64	16.22	35	
#17	M	69	1	LL	2.04	26.9	0.27	10.20	36	
#18	F	82	1	LL	2.77	21.3	0.23	10.78	32	
#19	M	44	0.5	UL, B	1.78	26.4	0.59	22.21	33	

#20	M	79	0.1	B	1.06	16.6	0.05	2.92	33	
#21	M	73	0.8	UL	1.50	12.4	0.10	8.09	30	
#22	F	80	0.8	B	0.55	30.2	0.32	10.67	27	
#23	M	50	0.5	UL	1.35	15.9	0.32	20.20	43	
#24	M	75	0.5	LL	1.88	638.7	2.04	3.19	34	Outlier
#25	F	86	0.5	B	1.22	211.4	1.76	8.31	35	

M, male; F, female; UL, upper limb; LL, lower limb; B, bulbar

Supplementary Table 2. Details of healthy volunteers for peripheral blood analysis in this study

Individual No.	Sex	Age (year)	Number of PBMCs ($\times 10^6$ cells/mL blood)	IP_6 level (pmol/10^6 cells)	IP_7 level (pmol/10^6 cells)	IP_7/IP_6 ratio ($\times 10^{-3}$)
#1	M	79	1.86	54.3	0.28	5.17
#2	F	57	3.56	26.4	0.52	19.82
#3	F	66	3.14	30.6	0.43	13.88
#4	F	66	3.11	30.2	0.56	18.65
#5	M	67	1.31	36.1	0.45	12.36
#6	M	72	3.07	30.0	0.33	10.85
#7	M	76	2.20	32.5	0.72	22.32
#8	M	82	1.90	72.7	0.85	11.64
#9	M	79	2.62	37.8	0.71	18.69
#10	M	76	1.44	31.2	0.04	1.34
#11	F	69	2.65	37.1	0.45	12.01
#12	M	74	1.66	83.5	1.14	13.70
#13	F	65	2.70	42.5	0.22	5.18
#14	F	65	3.33	27.0	0.26	9.68
#15	M	74	2.09	43.5	0.50	11.48
#16	M	77	2.70	48.9	0.60	12.22
#17	F	70	1.74	57.0	0.55	9.66
#18	F	63	1.27	60.1	0.60	10.01
#19	F	63	2.51	49.3	0.34	6.87
#20	F	63	1.89	59.4	0.41	6.95

#21	M	76	2.69	60.3	0.53	8.76
#22	M	69	1.76	66.7	0.19	2.89
